# Effect of combined ethanol and ultrasound pretreatment on energy consumption and bioactive compounds of rose petals in hot air drying

**DOI:** 10.1038/s41598-025-15820-0

**Published:** 2025-08-26

**Authors:** Mohammad Kaveh, Faroogh Sharifian, Esmail Khalife, Sasan Keramat, Behnam Ghaysari, Mahya Dolatkhoh, Faezeh Jadidi

**Affiliations:** 1 Department of Petroleum Engineering, College of Engineering, Knowledge University, Erbil, Iraq; 2 Department of Mechanical Engineering of Biosystems, Faculty of Agriculture, Urmia University, Urmia, Iran; 3 Department of Civil Engineering, Cihan University-Erbil, Kurdistan Region, Iraq; 4 Department of Biosystems Engineering, College of Agriculture and Natural Resources, University of Mohaghegh Ardabili, Ardabil, 56199-11367 Iran; 5 Department of Horticulture, Faculty of Agriculture, Urmia University, Urmia, Iran

**Keywords:** Medicinal plants, Essential oil, Ethanol, Brightness, Chemical engineering, Mechanical engineering

## Abstract

This study evaluates the impact of ultrasound (US), ethanol (ET), and combined ultrasound-ethanol (ET/US) pretreatments on the drying efficiency and quality of Rosa damascena petals during hot air drying. All pretreatments significantly reduced the drying time and specific energy consumption (p < 0.05). The highest reduction in drying time (52.38%) and specific energy consumption (64%) was observed for the ET/US pretreatment. Ultrasound alone (US30) and in combination with ethanol (ET/US) significantly improved rehydration ability and essential oil yield (up to 1.31%, p < 0.05). Furthermore, the ET/US treatment preserved more antioxidant activity, total phenolic content, and flavonoid content compared to other pretreatments and control (p < 0.05). The results of this study can show new perspectives for using the potential of ethanol- ultrasound as a cost-effective and energy-efficient method for industrial drying sensitive medicinal plants. Future studies could explore its scalability and applicability to other aromatic or perishable botanical species.

## Introduction

Medicinal plants have gained increasing attention for their bioactive compounds that contribute to therapeutic, nutritional, and aromatic properties^1^. Among them, rose (*Rosa damascena Mill*.) is extensively cultivated for its petals, which are valued for their essential oils used in cosmetics, perfumery, and even neuroprotective applications^2–4^. However, the high moisture content of fresh petals makes them extremely vulnerable to microbial spoilage, posing a challenge for post-harvest handling and storage^5–7^.

Drying is an effective method for preserving quality and extending shelf life by reducing moisture and inhibiting microbial growth^8–10^. Nevertheless, conventional drying methods, especially hot air drying, often compromise product quality by inducing aroma loss, nutrient degradation, structural damage, and high energy consumption^11,12^. These limitations have led to the exploration of novel pretreatment techniques aimed at improving drying efficiency while preserving sensitive compounds^13–16^.

Non-thermal pretreatments such as ethanol immersion and ultrasound have shown potential to enhance drying performance. Ultrasound waves (20–40 kHz) can disrupt plant tissue via cavitation and the sponge effect, forming microchannels that facilitate moisture transfer^17–19^. Tepe^20^ and Fotiou and Goula^21^ stated that ultrasound waves in the range of 20–40 kHz can be effective in improving quality characteristics, facilitating drying, and improving mass and heat transfer before drying various products. In addition, Boateng^22^ pointed out that ultrasound can create microchannels and cavitation bubbles by rapidly compressing and expanding food to release intracellular fluid to the surrounding area and rupture food tissue. Ultrasonic pretreatment was used to increase the drying speed of bitter melon^23^ and improve the rehydration ratio of ginger^11^ due to higher porosity. Shorter drying time, Inhibition of microbial growth, preservation of nutrients, properties of polyphenols, vitamins and carotenoids due to ultrasound have also been reported in the study^24–27^.

Ethanol, with its low boiling point, alters the membrane integrity and accelerates mass transfer through increased porosity and the Marangoni effect^28,29^. Together, these mechanisms facilitate faster water removal during drying while potentially reducing energy demands^30^. Recently, the combined use of ethanol/ultrasound processes has been developed as a promising, popular, rapid, economical and environmentally friendly pretreatment before the drying process, which relies on the ability of both methods to increase the quality of the final product and improve the drying process speed^9^. Ethanol softens and loosens the tissue matrix, while ultrasound amplifies moisture transfer through mechanical agitation—together offering a powerful pretreatment for enhancing drying performance and retaining sensitive compounds^31^. Martinez et al.^32^ and Miano et al.^33^ validated this phenomenon with a study of yacon potato and celery, respectively. Similarly, Rojas et al.^34^ investigated the impact of various pretreatment techniques on dried apples quality and found that the ethanol/ultrasound combination can reduce the drying time while improving the resorption coefficient and maintaining more antioxidant and phenolic content^34^. Furthermore, the use of ET/US in the pretreatment step has shown positive results such as reduced drying time, energy savings, improved water reabsorption coefficient, and greater preservation of nutritional properties of carrots^31^. This finding has also been confirmed on banana^16^ and ginger slices^35^.

To the best of our knowledge, there are considerable studies on drying roses by different drying methods in the literature. The effects of different drying methods such as hot air^2^, infrared^4^, comparison of different drying methods^5,36,37^, and the effects of different pretreatments before drying^38,39^ on the kinetics, energy, nutritional, qualitative, and physical properties of roses have been reported in the literature. Research investigating the use of the combined ultrasound/ethanol pretreatment effect for drying medicinal plants is still in its early stages, and there are no reports in the current literature on the effects of this drying method on different rose species. Furthermore, rose is a highly perishable medicinal plant with a high waste content, so using the combined ultrasound/ethanol method as a pretreatment before drying rose can be a challenge. This study aims to evaluate the effects of three non-thermal pretreatments—ultrasound (US), ethanol immersion (ET), and their combination (ET/US)—on the drying kinetics, energy efficiency, physicochemical quality, rehydration properties, bioactive compounds, and essential oil yield of *Rosa damascena* petals during hot air drying. This work introduces ET/US as a novel synergistic pretreatment, offering a promising approach to accelerate drying while preserving quality in sensitive medicinal plants.

## Materials and methods

### Plant material

In this study, rose with the geotype Rosa damascena Mill. L cultivated by the Horticulture Department—Urmia University, Iran in June 2024 was used. First, the petals were separated from other flower components in a cold place away from direct sunlight and then the samples were placed in special bags for preserving medicinal plants and stored in a refrigerator at 4 ± 1 °C until pretreatment and drying.

### Initial moisture content determination

Approximately 5 g of fresh petals were dried in a convection oven at 105 °C for 24 h. The initial moisture content of rose petals was determined to be 78 ± 0.2% (wet basis), following the AOAC standard method^40^, and calculated using Eq. (1)^30^.

1 $$MC = \frac{{M_{O} - M_{f} }}{{M_{f} }}$$

### Pretreatment procedures

#### Ultrasound pretreatment (US)

For ultrasonic treatment, the beaker containing 100 g of rose petals was placed in an ultrasonic bath (vCLEAN1-L2 model, manufactured by Beker Iran) containing distilled water in a ratio of 1:4 (w:v) at 30 °C. Ultrasound pretreatment was performed at a frequency of 25 kHz for two durations: 10 min (US10) and 30 min (US30). Each treatment was conducted in triplicate. Following sonication, excess surface water was gently removed using absorbent paper.

#### Ethanol pretreatment (ET)

Ethanol pretreatment was applied to the 100 g of rose petals in ethanol solutions for 10 min (ET10) and 30 min (ET30). For ethanol pretreatment, rose petal samples were immersed in ethanol solution at a 1:4 weight-to-volume ratio (w/v) and maintained at 25 °C in a water bath. After 10 (ET10) or 30 (ET30) minutes, the samples were removed and the surface moisture was gently blotted with filter paper.

#### Combined ethanol–ultrasound pretreatment (ET/US)

An ultrasonic bath (vCLEAN1-L2 model, manufactured by Beker Iran) with a frequency of 25 kHz was used for ET/US pretreatment. 100 g of rose petals were immersed in a glass beaker and an ultrasonic bath containing 400 ml of 100% ethanol for 20 min at 30 °C under ultrasonic waves. Then, the samples were removed from the ET/US and dried with paper.

#### Drying procedure

**Fig. 1:**
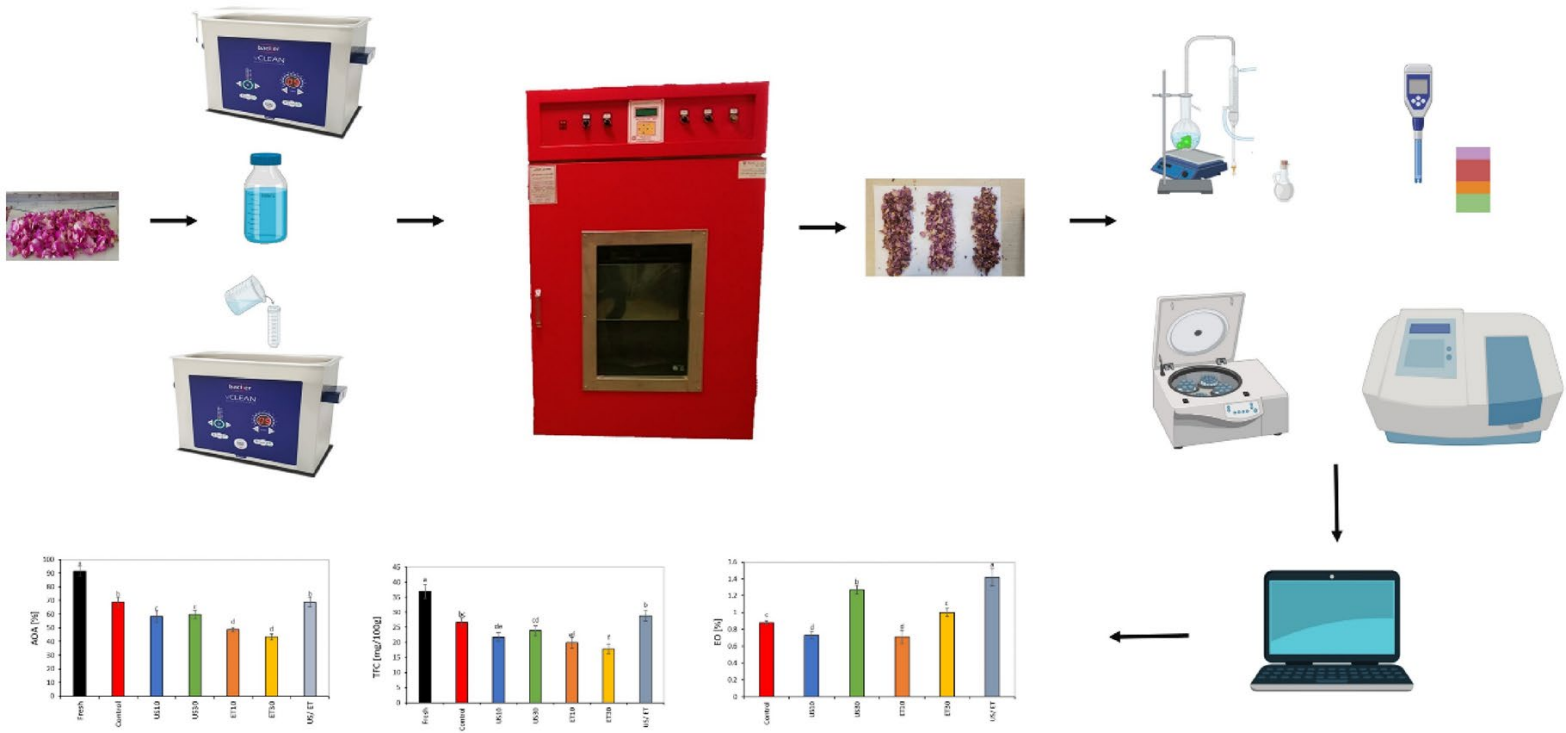
Illustrative diagram of rose petals preparation and analyses.

After exposing the petals to ET, US and ET/US pretreatments, all samples were dried with weights of 100 g in a hot air dryer (manufactured by Iran Grok Engineering and Design Company) at 50 °C and an air velocity of 1 m/s (for more details, see Sharifian et al.^41^). The weight reduction of the samples during the drying process was measured at each five minutes until reaching a moisture content of 0.1% on a dry basis using a precision balance in Excel software. Rose petal samples without pretreatment were also dried under hot air drying, resulting in a control treatment. Totally, 6 different treatments (1 control, 2 ethanol (ET10 and ET 30), 2 ultrasound (US10 and US30) and 1 ET/US) were tested. All experiments were repeated three times. Throughout the process, the measured average air temperature and relative humidity were 24.80 ± 1.27 °C and 43.45 ± 2.62%, respectively. After the drying test stage (pretreatment and hot air drying), the petals were stored at -5 °C until the physical, bioactive properties and essential oil yield tests were performed. The way the tests were performed is shown in Fig. 1.

#### Drying kinetics, drying rate and mathematical modeling

How the moisture ratio of rose petals changed during drying for each pretreatment was investigated using Eq. (2). The time required for the product to reach 10% moisture content on a wet basis for each treatment was measured. After that, the drying rate of the samples for each pretreatment was measured using Eq. (3)^23,42^.

2 $$MR = \frac{{M_{t} - M_{e} }}{{M_{o} - M_{e} }}$$

3 $$DR = \frac{{M_{t + dt} - M_{t} }}{\Delta t}$$

To analyze and predict the drying behavior of rose petals under different pretreatments, various thin-layer drying models (Table 1) were applied. Moisture ratio data were plotted against drying time, and the experimental data were fitted to each model. Model performance was assessed using statistical indicators: coefficient of determination (R^2^), chi-square (χ^2^), and root mean square error (RMSE), as defined in Eqs. (4)–(6)^43^.

4 $$R^{2} = 1 - \frac{{\sum\nolimits_{i = 1}^{N} {\left[ {MR_{pre,i} - MR_{real,i} } \right]^{2} } }}{{\sum\nolimits_{k = 1}^{N} {\left[ {MR_{pre,mean} - MR_{real,i} } \right]^{2} } }}$$

5 $$RMSE = \left[ {\frac{1}{N}\sum\limits_{i = 1}^{N} {\left( {MR_{pre,i} - MR_{real,i} } \right)^{2} } } \right]^{\frac{1}{2}}$$

6 $$\chi^{2} = \frac{{\sum\nolimits_{i = 1}^{N} {\left( {MR_{real,i} - MR_{pre,i} } \right)^{2} } }}{N - z}$$

**Table 1:** Mathematical models applied to drying curves of rose petals.

Model name	Model formula	References
Logarithmic	$MR = a\exp ( - kt) + c$	Akhoundzadeh Yamchi et al.^23^
Parabolic	$MR = a + bt + ct^{2}$	Tepe^30^
Page	$MR = \exp ( - kt^{n} )$	Zubernik et al.^44^
Yuan	$MR = \frac{{a + bt + ct^{2} }}{{1 + dt + ft^{2} }}$	Yun et al.^45^
Henderson and Pabis	$MR = a\exp ( - kt)$	Salehi et al.^46^
Weibull	$MR = \exp \left[ { - \left( \frac{t}{b} \right)^{a} } \right]$	Duan et al.^47^
Two-term	$MR = a\exp ( - kt) + c\exp ( - k_{0} t)$	Oladejo et al.^8^

#### Effective moisture diffusion

Fick’s second law was used to determine the moisture diffusion. The second law of Fick’s diffusion equation, which implies the diffusion of mass during the period of decreasing rate of drying of agricultural products, is shown in Eq. 7^48^.

7 $$\frac{\partial x}{{\partial t}} = D_{eff} \frac{{\partial^{2} x}}{{\partial x^{2} }}$$

Solving the diffusion equation by Crank, assuming uniform initial moisture, constant diffusion coefficient, ignoring temperature fluctuations and shrinkage of the product during drying^49^, leads to Eq. 8^20^.

8 $$MR = \frac{{M_{t} - M_{e} }}{{M_{o} - M_{e} }} = \frac{8}{{\pi^{2} }}\sum\limits_{n = 1}^{\infty } {\frac{1}{(2n + 1)}} \exp \left( {\frac{{ - D_{eff} (2n + 1)^{2} \pi^{2} t}}{{4L^{2} }}} \right)$$

Finally, using Eq. (9), the D_eff_ is obtained

9 $$D_{eff} = \left[ {\frac{{\pi^{2} D_{eff} }}{{4L^{2} }}} \right]$$

#### Specific energy consumption (SEC)

Equation (10) was used to calculate the energy consumption (SET) during the drying process of rose petals in a hot air dryer^50^.

10 $$SET_{{con}} = A.v.\rho .t_{c} .C_{{pa}} .\Delta t$$

In addition, the ultrasound energy consumption was calculated from Eq. (11)^35^.

11 $$SET_{US} = W \times V \times t_{p}$$

Specific energy consumption (SEC), defined as the energy required to remove 1 kg of water from rose petals, was calculated for each pretreatment method and hot air drying process using Eq. (12)^51^.

12 $$SEC = \frac{{SET_{US} + SET_{con} }}{{M_{w} }}$$

### Quality analyses

#### Color

The color values of rose petals were measured using a colorimeter (FRU WR10, China) before and after the drying process (including pretreatment and drying). To obtain accurate values based on L*a*b*, color measurements were performed at three different points on the surface of the samples. The total color difference (ΔE) between the color values of the fresh and dried product was calculated using Eq. (13)^35^. The ΔE value indicates the magnitude of the color change, where higher values indicate a significant color change during the drying process.

13 $$\Delta E = \sqrt {(L_{0}^{*} - L^{ * } )^{2} + (a_{0}^{*} - a^{ * } )^{2} + (b_{0}^{*} - b^{ * } )^{2} }$$

#### Water activity

The Water activity of rose samples before and after drying was determined by an instrument (LabMaster, Switzerland) at 25 °C.

#### Rehydration ratio

The resorption coefficient property is used to assess the degree of cell damage of different products during drying. The method for determining RR was based on the method of Kian-Pour et al.^52^ with some modifications. Accordingly, about 2 g of dried rose petals were enclosed in a special mesh and immersed in distilled water at 25 °C for two hours. The petal/distilled water ratio was about 1:20. Then the sample was drained with a filter paper and weighed. RR was calculated as follows^30^.

14 $$RR = \frac{{m_{r} }}{{m_{d} }}$$

### Bioactive analyses

#### Extraction

The extract from rose petals was prepared according to the method described by Cruz et al.^51^. For this purpose, 0.3 g of petals were mixed in special plastic tubes containing 30 ml of methanol (50%), stirred for half an hour at 25 °C. Then, the prepared samples were placed in an ultrasonic bath for half an hour and the supernatant obtained was placed in a special tube. Then, 30 ml of acetone (70%) was added to it and stirred again. The supernatant obtained was added to the first solution. After that, distilled water was added to the extract until its final volume reached 50 ml.

#### Antioxidant activity

The antioxidant activity was evaluated by DPPH assay according to the method described by Granella et al.^53^ with minor modifications. According to their method, 1 ml of the extract was transferred to test tubes containing 3 ml of DPPH radical solution and stirred for 30 min. Then, the new solution was measured by spectrophotometer at 515 nm after two hours in a dark room. The results were calculated in percent inhibition (%) based on Eq. (15)^48^.

15 $$AOA = \frac{{A_{c} - A_{d} }}{{A_{c} }} \times 100$$

#### Total phenolic content

The total phenolic content of rose petals before and after drying was determined according to the Folin-Ciocalteu method and according to the method of Altay et al.^54^. 40 μl of the prepared extract was combined with 3.16 ml of distilled water and 0.25 ml of Folin-Ciocalteu reagent in a laboratory flask and stirred for 10 min. Then, 1.5 ml of sodium carbonate (10%) was added to the laboratory flask containing the solution and stirred again for 5 min. Then, the final mixture was kept at 25 °C for two hours and finally the phenolic content was measured with a spectrophotometer at a wavelength of 765 nm.

#### Total flavonoid content

The total flavonoid content was estimated based on the aluminum trichloride colorimetric method and according to the study of Zamani et al.^10^. 100 μL of petal powder extract was combined with 75 μL of sodium nitrite (0.5 M) and 75 μL of aluminum chloride (0.3 M) in a laboratory flask and then immersed in a certain amount of distilled water. In the following operation, about 500 μL of NAOH (1 M) was added to the solution in the flask. The final prepared solution was placed in absolute darkness for two hours and then tested using a spectrophotometer at a wavelength of 510 nm.

#### Essential oil yield

The method of Nalawade et al.^12^ was used to determine the essential oil yield of rose petal samples. Therefore, 20 g of dried samples were distilled with distilled water in a Clevenger apparatus for 180 min. The EO yield was evaluated with Eq. (16)^10^:

16 $$EOY = \frac{{E_{w} }}{{W_{s} }} \times 100$$

## Results and discussion

### Moisture ratio

**Fig. 2:**
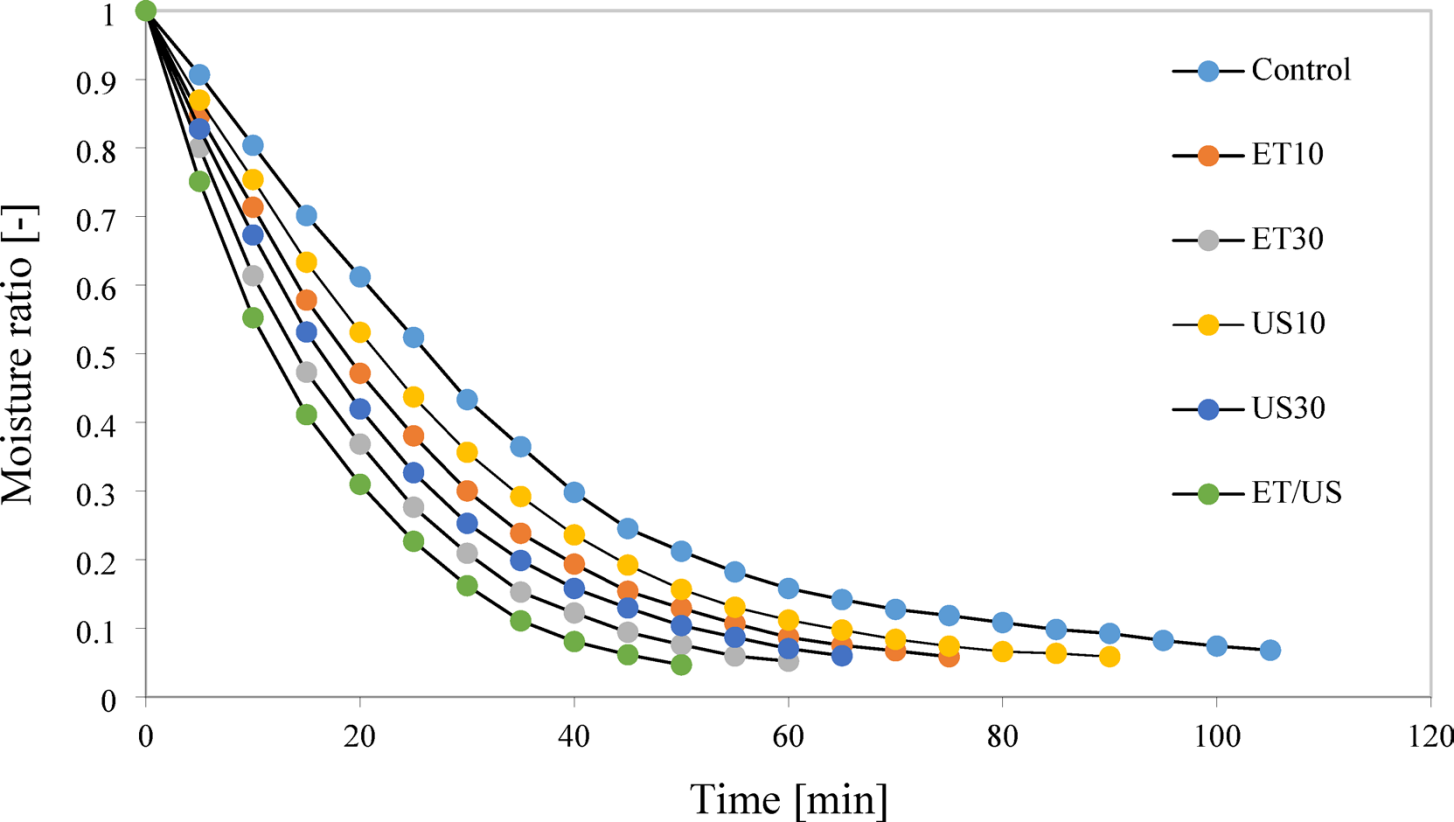
Drying kinetics of dried rose petal under different drying pretreatments.

**Fig. 3:**
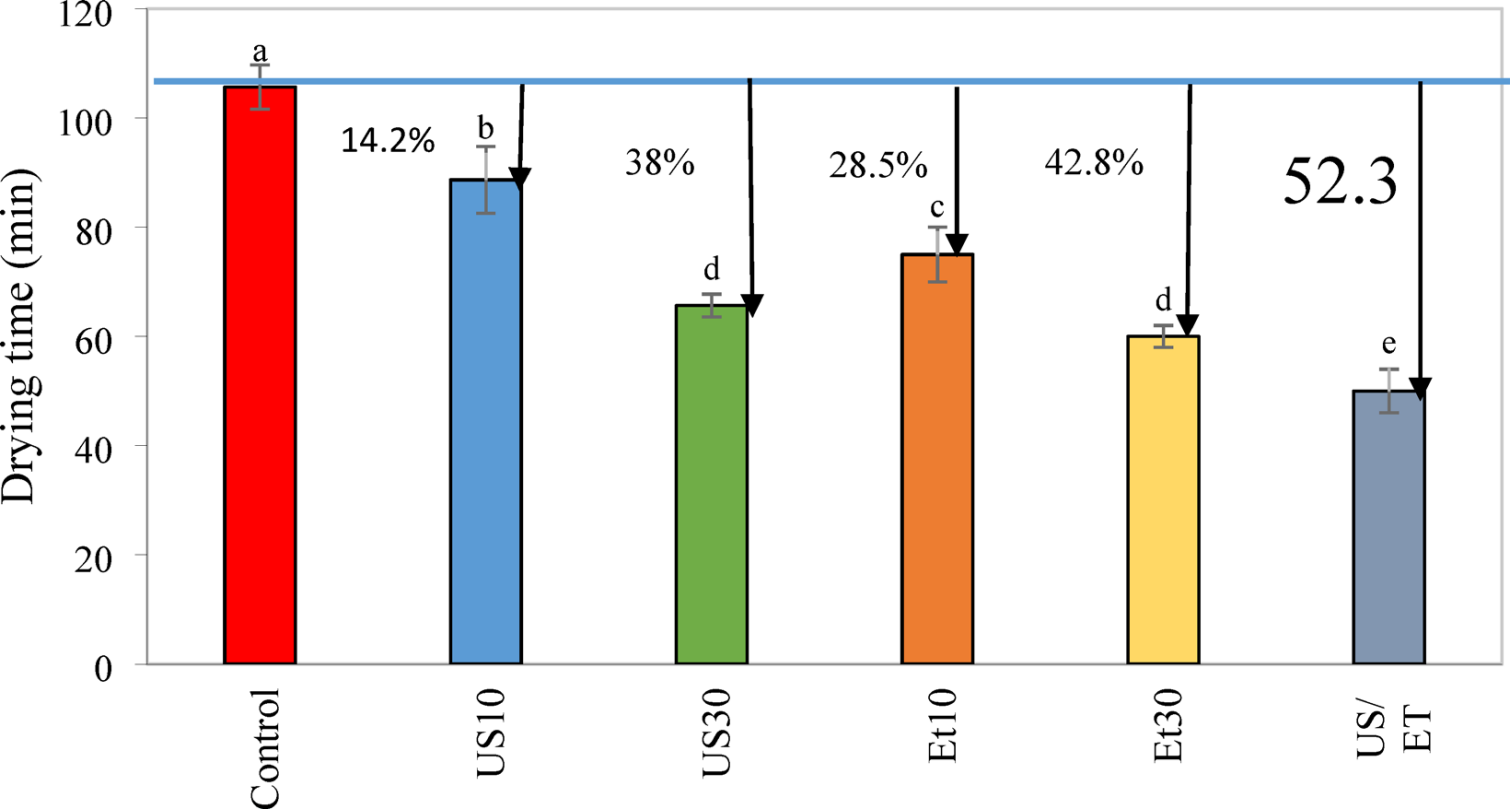
Reduction of the drying time for each drying pretreatment of the rose petal. Vertical bars are the standard deviation obtained from the four replicates. Different letters indicate significant differences among treatments.

The dimensionless moisture ratio (MR) of rose petals versus drying time under different pretreatments is shown in Fig. 2. The drying process was stopped when the sample weight changes in the last three weights became almost constant. According to Fig. 2, the MR of the samples for all pretreatments decreased exponentially with the progress of drying. The average values of drying time under different pretreatments are shown in Fig. 3. Based on the results obtained from Fig. 3, about 28.5% reduction in drying time was observed for ethanol for 10 min and 42.8% for ethanol for 30 min. Ethanol, due to its “Marragoni effect”, promotes a surface tension gradient at the two water/ethanol interface, faciliating the mass transfer phenomenon occurs faster with the thinning of the cell wall^51^. In addition, it dissolvs some cell wall components, eliminating intercellular air and creating new pathways for faster water transport^9^. This reduction in time during convective drying with ethanol pretreatment in previous studies is consistent with that observed in previous studies. For examples, Rojas et al.^50^ reported that using ethanol pretreatment for 30 and 10 min reduced the drying time by 52% and 49% for pumpkin, respectively. Granella et al.^35^ also noted that same pretreatment for 10 min reduced the drying time by 13.7% for bananas in a hot air dryer, while this reduction was 13.2% in 5 min ethanol pretreatment.

Ultrasound treatments US10 and US30 reduced drying time by 14.2 and 38%, respectively, compared to conventional drying (control sample). The drying rate was higher in samples pretreated with US30 than US10. This reduction in drying time with ultrasound has been previously reported in the literature. For example, 16% reduction in drying time was observed for ginger^11^, 35% for faba beans^46^, 46% for roselle^8^. Ultrasound waves, due to acoustic cavitation, cause a spongy effect in the product, which can lead to the creation of microchannels within the food sample^32^. Kian-Pour et al.^52^ emphasized that the increased formation of microchannels and spongy effect during 30 min of pretreatment application contributed to faster water transfer of the food sample.

Combined ethanol/ultrasound pretreatments further reduced drying time by 38.52%, similar to that achieved by using ethanol/ ultrasound pretreatment. For example, 45.6% time reduction was reported for scallion stalk slices immersed in ethanol/ ultrasoun^29^, 9.8% to 18.3% for apples treated under different conditions with ethanol/water^44^, and 49.3% for papayas subjected to ethanol/ultrasound pretreatmen^13^. Compared to ET or US applied separately, greater drying time reduction was achieved with the combination of ethanol/ultrasound pretreatments during drying. Therefore, ET/US pretreatments shorten the drying time by 33.3, 6.16, 4.44 and 07.23% compared to ET10, ET30, US10 and US30, respectively. The effects of thinning and rupture of cell walls, removal of intercellular air and reduction of resistance to fluid flow due to surface tension-driven mass transfer with ethanol (ET)^32^ and the formation of microchannels and reduction of boundary layer thickness due to acoustic cavitation (US effect) improve water flow by capillary action^50^. Ren et al.^16^ reported that the use of ethanol/water mixture dried ginger by 31% and 41.3% faster compared to ethanol and ultrasound alone, respectively. In addition, Amanor-Atiemoh et al.^55^ showed that for drying apples under 60 °C, the ethanol/ultrasound mixture can reduce the drying time by 16.6 and 9% compared to ultrasound and ethanol, respectively.

### Drying kinetics modeling

**Fig. 4:**
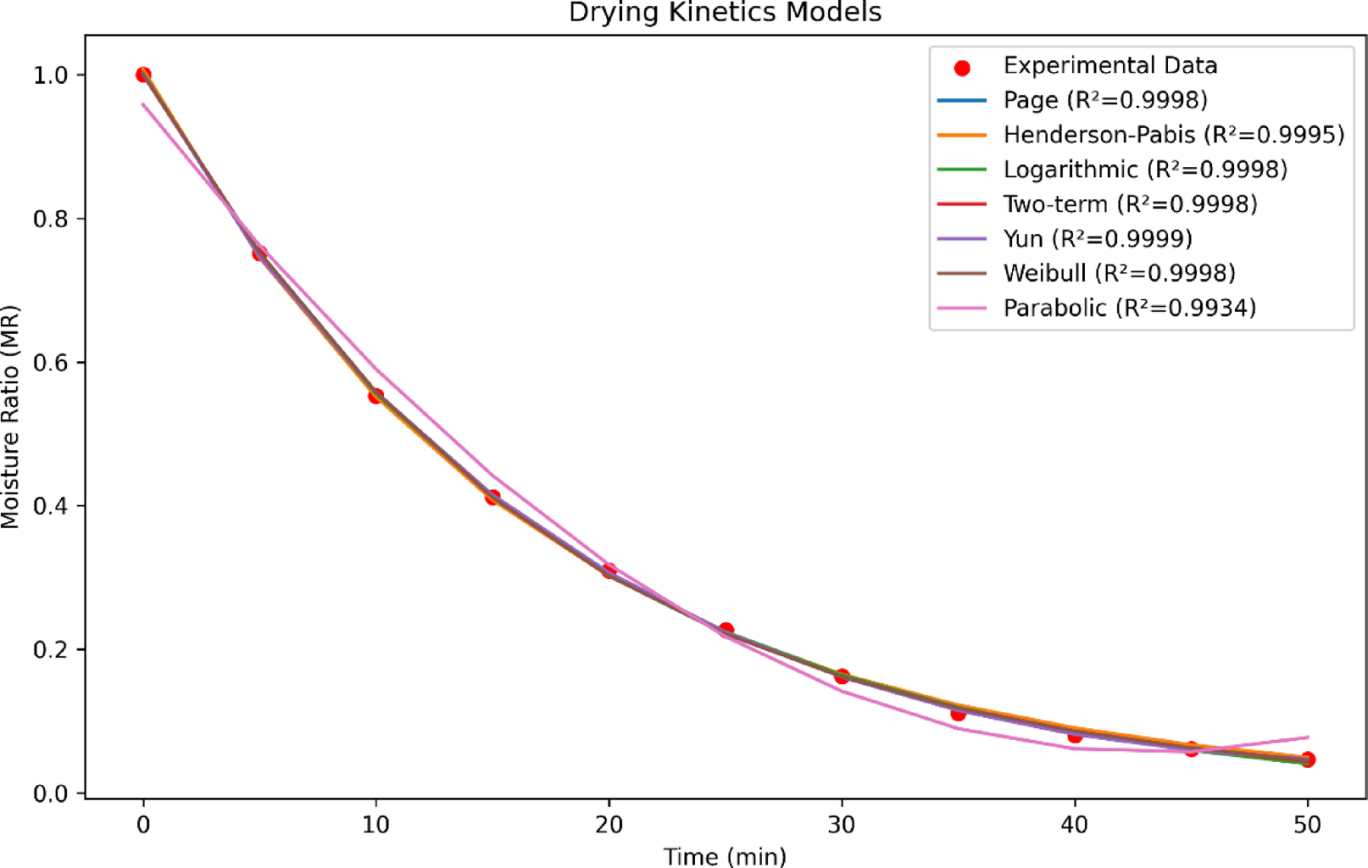
Drying curves of the rose petals modelled by different mathematical model in ET/US pretreatment.

The aim of modeling is to predict drying kinetics under all drying conditions while observing the model performance range. The number of model parameters mentioned in Table 1 was fitted with the drying kinetics under each pretreatments using MATLAB 2021 software. The values of RMSE, χ^2^ and R^2^ results of fitting the curves with different models in different pretreatments are listed in Table 2. Accordingly, it is evident that in all models related to drying under different pretreatments, the R^2^ higher than 0.9919 was obtained, so that the Yun et al. model has the highest R^2^ (0.9996–1) and the lowest RMSE (0.0056–0.0012) and χ^2^value (0.000033–0.000003); therefore, this model was chosen as the most suitable for describing and predicting the drying process of rose petals with the help of hot air for all pretreatments. The fit of different models to experimental data for all pretreatments (in ET/US) is clearly seen in Fig. 4. In addition, the constant coefficients of all models for all pretreatments are reported in Table 2.

**Table 2:** Results of statistical analysis of each mathematical model of rose petals under different pretreatment conditions.

Model	Drying method	R^2^	RMSE	χ^2^	k	n	a	b	c
Henderson and Pabis	Control	0.9942	0.0219	0.00052	1.043	0.0293			
	ET10	0.9980	0.0129	0.00019	1.026	0.0401			
	ET30	0.9991	0.0087	0.00009	1.057	0.0515			
	US10	0.9968	0.0166	0.00033	1.023	0.0438			
	US30	0.9985	0.0111	0.00014	1.021	0.0450			
	US/ET	0.9995	0.0063	0.00005	1.003	0.0602			
Page	Control	0.9949	0.0203	0.00045	0.018	1.1063			
	ET10	0.9991	0.0083	0.00019	1.026	0.0401			
	ET30	0.9996	0.0056	0.00003	0.042	1.0564			
	US10	0.9984	0.0098	0.00038	0.055	1.0945			
	US30	0.9993	0.0073	0.00006	0.034	1.0735			
	US/ET	0.9997	0.0042	0.00002	0.053	1.0352			
Logarithmic	Control	0.9943	0.0217	0.00051	1.038	0.0301	0.0091		
	ET10	0.9981	0.0124	0.00017	1.034	0.0387	-0.013		
	ET30	0.9992	0.0083	0.00008	1.021	0.0504	-0.008		
	US10	0.9969	0.0166	0.00042	1.036	0.0362	-0.010		
	US30	0.9986	0.0107	0.00013	1.028	0.0437	-0.106		
	US/ET	0.9998	0.0041	0.00002	1.018	0.0576	-0.016		
Parabolic	Control	0.9933	0.0232	0.00061	0.990	-0.021	0.0001		
	ET10	0.9960	0.0183	0.00038	0.975	-0.028	0.0002		
	ET30	0.9939	0.0229	0.00061	0.963	-0.035	0.0003		
	US10	0.9956	0.0195	0.00048	0.968	-0.033	0.0002		
	US30	0.9956	0.0192	0.00043	0.974	-0.032	0.0002		
	US/ET	0.9933	0.0243	0.00072	0.958	-0.041	0.0004		
Weibull	Control	0.9949	0.0203	0.00045	35.98	1.106			
	ET10	0.9991	0.0083	0.00007	25.90	1.087			
	ET30	0.9996	0.0056	0.00003	19.86	1.056			
	US10	0.9984	0.0102	0.00014	30.25	1.099			
	US30	0.9993	0.0073	0.000061	22.93	1.072			
	US/ET	0.9997	0.0042	0.000022	16.81	1.035			
Yun	Control	0.9996	0.0056	0.000033	0.996	-0.014	0.00009	0.0010	0.0005
	ET10	0.9999	0.0022	0.000005	0.998	-0.019	0.00013	0.0100	0.0005
	ET30	0.9998	0.0033	0.000013	1.001	-0.024	0.00018	0.0194	0.0005
	US10	1.0000	0.0012	0.000009	0.998	-0.016	0.00008	0.0133	0.0006
	US30	0.9999	0.0016	0.000003	0.999	-0.018	0.00012	0.0149	0.0008
	US/ET	0.9998	0.0030	0.000011	1.001	-0.033	0.00031	0.0258	-0.0001
Two-term	Control	0.9942	0.0219	0.00052	0.447	0.029	0.5955	0.0293	
	ET10	0.9980	0.0129	0.00019	0.505	0.040	0.5204	0.0401	
	ET30	0.9991	0.0087	0.00009	0.505	0.051	0.5098	0.0515	
	US10	0.9972	0.0168	0.00024	0.482	0.035	0.5662	0.0354	
	US30	0.9985	0.0111	0.00014	0.648	0.045	0.3734	0.0450	
	US/ET	0.9995	0.0063	0.00005	0.485	0.060	0.5214	0.0602

### Drying rate

**Fig. 5:**
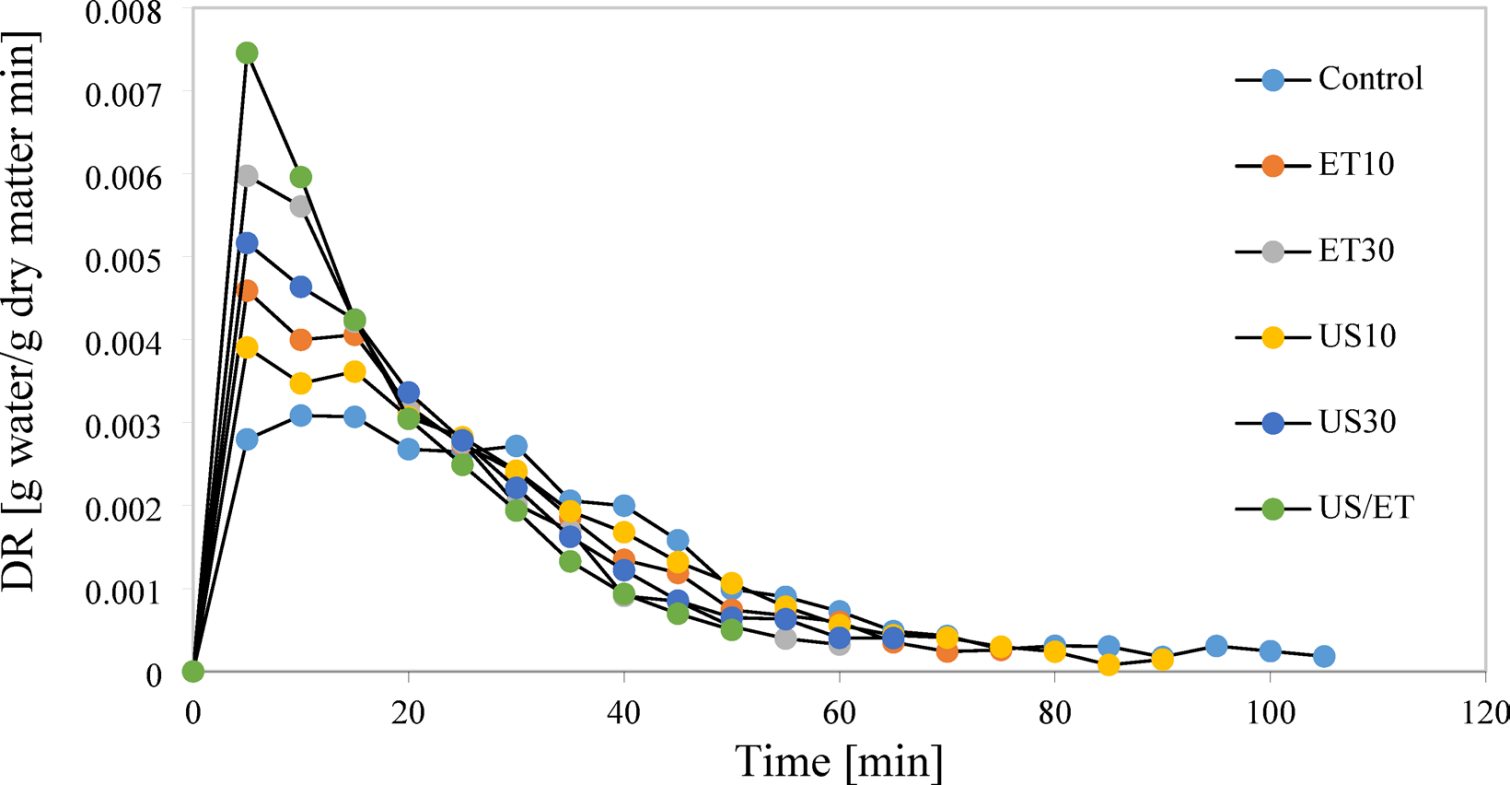
Drying rate with and without pretreatment.

Figure 5 shows the impact of ultrasound, ethanol and ultrasound/ethanol pretreatments on the drying rate of rose petals versus time. All treatments showed a decreasing drying rate proportional to time. Initially, moisture was removed rapidly due to weak bonding and minimal internal resistance^48^. However, at the end of this stage and upon entering the descending stage, all the free water in the samples evaporated and, due to its resistance to moisture removal, the drying rate of the product decreased^43^.

Increasing the duration of the ultrasound pretreatment and the ethanol application time increased the drying rate of the petals due to facilitating moisture removal. However, the effect of the ethanol/ultrasound combination on the drying rate of petals was greater than that of ethanolized and sonicated samples. Because the simultaneous effect of ethanol/ultrasound leads to the destruction of the cell structure due to acoustic cavitation^31^ and thinning of the cell wall and greater permeability due to the Internal moisture movement due to surface tension changes^32^. The drying rate trend with the constant drying rate data (k value) presented in Table 2 is as Control < US10≈ET10 < US30 < ET30≈ET/US. Similarly, higher drying rates were reported in peach samples immersed in ethanol/ultrasound compared to control, ultrasound and ethanol samples by Fotiou and Goula^21^. Similar findings were also reported by da Silva et al.^56^ in grape, Kian-Pour et al.^52^ in beetroot, and Zhou et al.^29^ in scallion stalk slices drying process, where ET/US treatments consistently surpassed individual pretreatments.

### Effective moisture diffusivity (D_eff_)

The diffusion coefficient of the rose samples under different pretreatments ranged from 4.48 × 10^–9^ to 9.56 × 10^–9^ m^2^/s (Table 3), falling within the common range for majority food materials, i.e. 10^–12^ to 10^–7^ m^2^/s. All pretreatments of US, ET, and ET/US significantly increased D_eff_ compared to the control, which increased the moisture transmitting to the surface layers of the samples. While the control sample showed the minium value. Among the different treatments, the D_eff_ of the E10 and E30 samples was more than that of the US10 and US30 samples, likely due to the breakdown of the cell wall by using ethanol and the creation of new channels for moisture movement.

**Table 3:** The range of the D_eff_ calculates by Fick’s second law model under different drying pretreatments.

Treatment	D_eff_ × 10^–9^ (m^2^/s)	R^2^
Control	4.48	0.9789
US10	5.75	0.9958
US30	7.50	0.9972
ET10	6.71	0.9886
ET30	8.61	0.9977
US/ET	9.56	0.9997

On the other hand, by increasing the ultrasonic pretreatment time before hot air drying from 10 to 30 min, the D_eff_ value improved. At higher US, ultrasonic waves induced cavitation, reduced internal resistance, expansion and further compression in the rose tissue^8^. The positive contribution of ultrasonic pretreatment on the D_eff_ value was observed by Zang et al.^19^ Akhoundzadeh Yamchi et al.^23^, Abbaspour-Gilandeh et al.^57^ and Salehi^58^ for *Angelica sinensis*, bitter melon, cantaloupe and mung beans, respectively.

Meanwhile, increasing the ET pretreatment time from 10 to 30 min increased the D_eff_ value. Tissue rupture, damage to boundary layers, formation of micropores, and formation of turbulent water flow caused by dissolution of certain cell wall components in ethanol lowered the external resistance to moisture migration in the samples. Therefore, this ethanol flow accelerates the diffusion and evaporation of water and improves moisture diffusion. Other studies support the effect of ethanol increasing D_eff_ on various foods, which has been reported by Santos et al.^59^, Rojas et al.^60^ and Tepe and Tepe^61^.

In addition, the effect of ET/US pretreatment let to a the synergistic interaction surpassing the individual effects of each method. Fotiou and Goula^21^ reported that ET/US before drying notably enhanced the D_eff_ of peach samples compared to untreated, ethanol, and ultrasound-treated samples, as reported in the study of Granella et al.^35^.

### Energy consumption

Drying is a costy operation and energy-intensive – process in the food industry, which has adverse effects on the environment. Therefore, the use of new technologies to reduce energy and increase energy efficiency is very important^62,63^. The reduction of the drying process time (pretreatment-hot air) discovered interesting resulton the energy consumption reduction. The total energy consumption (SEC) and the percentage of energy reduction for each pretreatment during the hot air drying process of rose petals (to a final moisture content of 10% on a wet basis) were estimated with Eq. (12). Table 4 represents an estimate of the comparison of the average total energy consumption (TEC) and the percentage of energy reduction. The energy consumed for drying the samples using ethanol, ultrasound and ethanol/ultrasound was highly reduced. The specific energy consumption values for ET10 and ET30 were 7.06 and 5.64 MJ/kg, respectively, resulted in a reduction of 44.84 and 55.9% in energy consumption compared to the control sample. According to Silveira et al.^64^, the Marangoni effect and changes in cell wall thickness caused by ethanol reduced energy consumption. Interestingly, during pretreatments US10 and US30 showed 9.38 and 8.08 MJ/kg, respectively, which were 26.71 and 37.42% less than the control sample, respectively. SImilar findings are reported in literature. Silveira et al.^62^, obtained a 34.41% reduction in energy consumption for yacon, Tepe^30^ a 25% to 37.50% reduction for carrots, and Cruz et al.^51^ a reduction of 15% for drying papaya with ethanol. Ghanbarian et al.^7^ obtained a 9.03% to 15.8% reduction in SEC at 50°C for peppermint leaves with US. Also, a reduction in SEC with US pretreatment was reported in the study of Santos et al.^65^ for drying avocado.

**Table 4:** Energy consumed for each convective drying pretreatment of the rose petals. Note: Values with the same superscript are not significantly different (p > 0.05).

Pretreatment	Energy consumption: Ultrasound (ET_US_) (MJ/kg)	Energy consumption: Drying (ET_con_) (MJ/kg)	Total energy consumption (SEC) (MJ/kg)	Reduction of energy consumption (%)
Control		12.81	12.81 ± 0.83^a^	-
US10	1.18	8.19	9.39 ± 0.83^b^	26.71
US30	2.08	5.92	8.02 ± 0.83^c^	37.42
ET10	-	7.06	7.06 ± 0.83^d^	44.84
ET30	-	5.64	5.64 ± 0.83^e^	55.90
ET/US	2.08	2.51	4.61 ± 0.83^f^	64

As shown in Table 4, samples immersed in the combined ET/US pretreatment recorded the lowest SEC (4.61 MJ/kg) and the highest reduction in SEC (64%) compared to the control. Furthermore, compared to ET10, ET30, US10 and US30, the ET/US pretreatment showed a reduction of 34.8, 18.40%, 50.9% and 42.50%, respectively. Therefore, the use of ET/US pretreatment provides a significant reduction in SEC compared to other processes (P < 0.05). In summary, the combined ET/US pretreatment was more efficient in drying rose petals than the other methods, which was due to structural changes in the cells, a reduction in internal and external resistance caused by the Marangoni effect and the sponge effect resulting from the synergy of both pretreatments^31^. Similar results of reduced SEC from the ET/US combination compared to ethanol and ultrasound alone have been reported by Santos et al.^31^, Rojas et al.^50^ and Granella et al.^35^.

It is important to emphasize that a decrease in SEC does not always lead to a lower total production cost. The estimated expenses including equipment (US, ET and dryer), energy and raw materials depend on the socio-economic and geographical conditions of each region worldwide. However, the reduction in energy consumption in itself is a highly desirable achievement for scientific, social and environmental contributions.

### Water activity (aw)

**Fig. 6:**
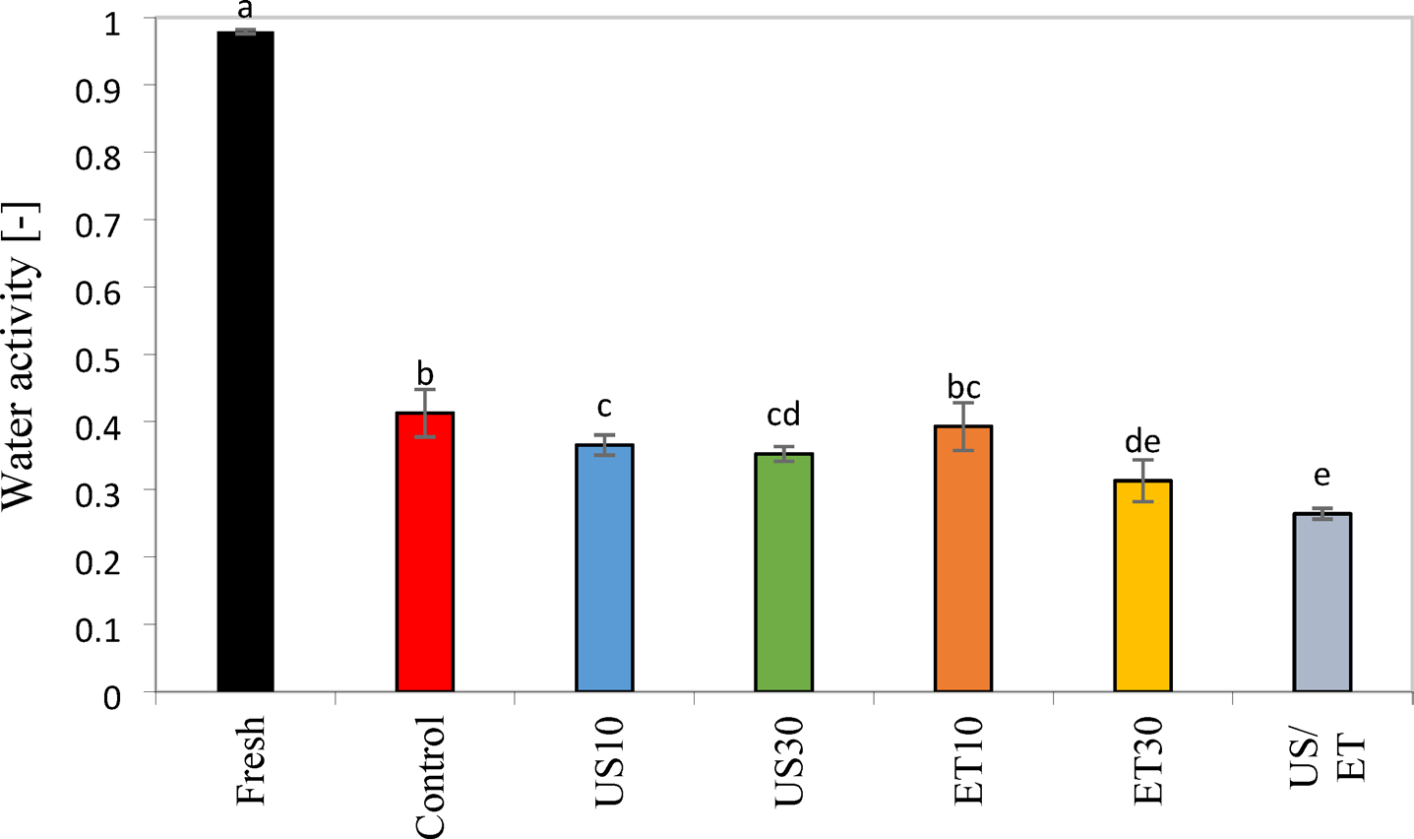
Water activity for fresh and with and without pretreatment of the samples. The same letter indicates no significant difference among the treatments, according to Tukey’s test (p < 0.05).

Rose petals are highly perishable mainly due to their high aw. Therefore, drying can be a technique used for its preservation^13^. The average aw values under different conditions are reported in Fig. 6. The aw of fresh rose samples was similar to that reported by Hnin et al.^6^. According to Fig. 6, all dried petals—with and without pretreatment—showed significantly lower aw values compared to the fresh sample, all being below 0.60. At aw values below 0.6, conditions are unfavorable for the growth of pathogenic bacteria and fungi^66,67^. However, samples dried with pretreatments (ET/US, ET and US) showed lower values of aw compared to samples dried with a hot air dryer. No significant difference was observed between different ultrasound times. However, the ET/US combination significantly reduced aw compared to the other pretreatments (p < 0.05), because the ET/US solvent increases the permeability of the cell wall and facilitates water removal^64^.

### Color

**Fig. 7:**
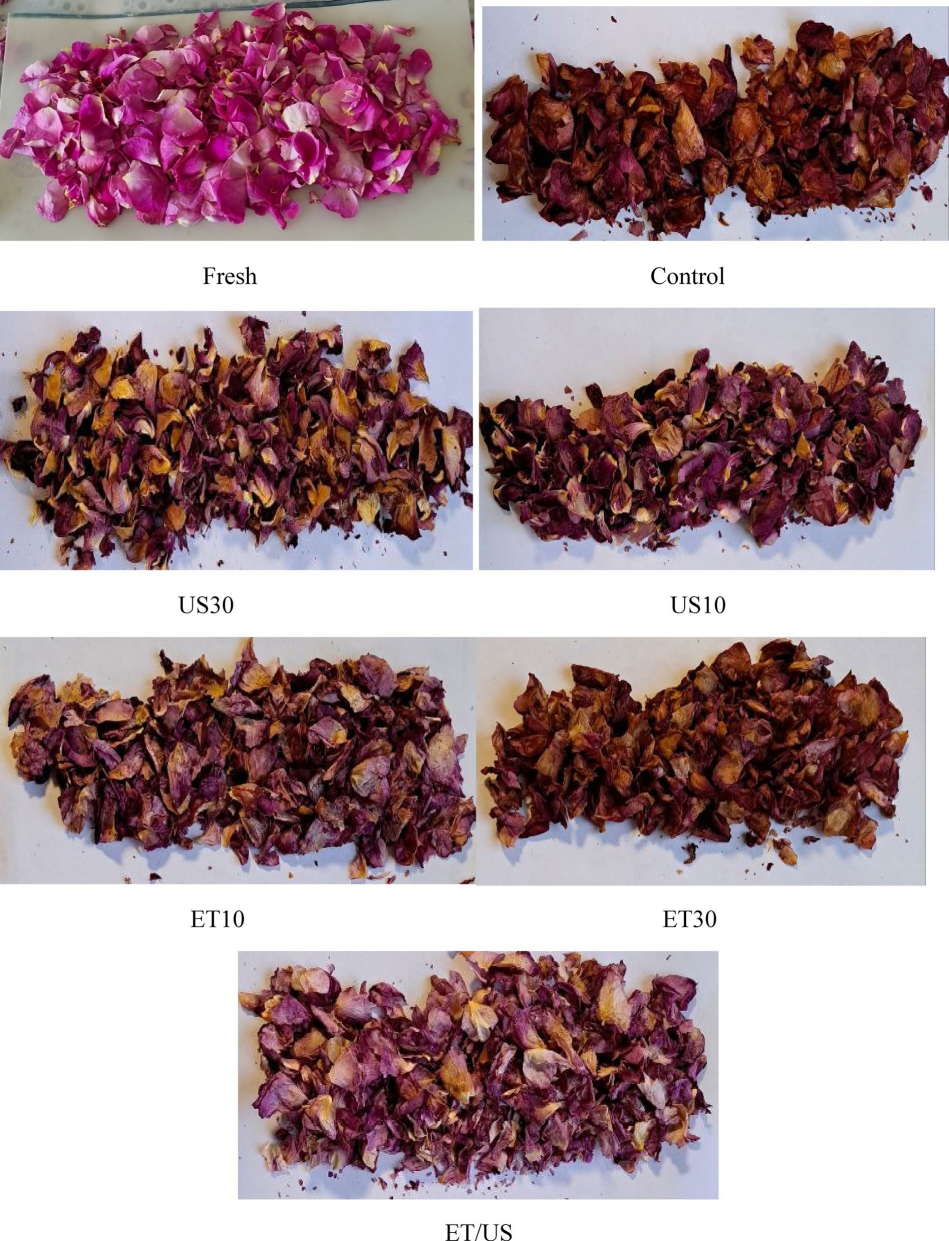
Rose petals samples before and after the hot air drying process in different pretreatment.

The average values of color parameters L, a, b and ΔE of rose petals dried under different conditions of ethanol, ultrasound and ethanol/ultrasound pretreatment are shown in Table 5. The values of L*, a*, b of fresh roses were 40.65, 14.86 and 1.78. As is shown, the effect of all pretreatments on the values of L*, a* and b* was significant (p < 0.05). According to Guo et al.^68^, the higher the brightness degree (L*), the lower the browning reaction will be. According to the images of dried rose samples (Fig. 7), it can be observed that the color of dried samples after ET/US pretreatment was lighter than other treatments. This may be because the synergistic effect of ET/US prevents browning caused by enzymatic reactions. In addition, the minimum L* value measured was obtained for ET30. Non-enzymatic browning and enzymatic reactions during prolonged immersion in ethanol may cause more browning in the sample treated with ET30, resulting in a greater decrease in L* value than other treatments. On the other hand, pretreatments increased the a* value, which was the highest for ET30. This indicates that the color of roses after ET30 becomes darker and redder compared to other pretreatments. Barani et al.^69^ showed that rose samples with higher a* and b* are darker and redder. No clear trend was observed for b* across treatments while b* increased in some pretreatments such as control, ET10, ET30 and ethanol/ultrasound compared to fresh sample, but samples pretreated with US10 and US30 decreased b* compared to fresh sample.

**Table 5:** The color parameters of dried rose petals under different drying pretreatments. Note: Values with the same superscript are not significantly different (p > 0.05).

Pretreatment	L*	a*	b*	ΔE
Fresh	40.65 ± 0.70^a^	14.86 ± 0.32^a^	1.78 ± 0.12^cd^	-
Control	31.36 ± 0.78^d^	19.22 ± 0.72^d^	4.11 ± 0.13^a^	10.53 ± 1.07^c^
US10	34.26 ± 0.55^c^	21.54 ± 0.91^c^	1.39 ± 0.21^de^	9.50 ± 0.52^c^
US30	35.84 ± 2.30^bc^	19.78 ± 0.83^d^	1.02 ± 0.55^e^	7.09 ± 0.41^d^
ET10	28.51 ± 0.53^e^	23.30 ± 0.90^b^	3.64 ± 0.30^ab^	14.89 ± 1.37^b^
ET30	25.48 ± 1.10^f^	24.88 ± 0.55^a^	3.50 ± 0.39^b^	18.28 ± 0.77^a^
ET/US	36.70 ± 0.34^b^	21.84 ± 0.32^c^	2.23 ± 0.20^c^	7.79 ± 0.43^d^

ΔE, indicating overall color variation. The ΔE values of the samples were 10.53, 9.50, 7.09, 14.89, 18.28 and 7.79 for control, US10 and US30, ET10, ET30 and ET/US, respectivbely. Notably, the ΔE values for US30 and ET/US were comparable, whereas ET30 showed remarkable higher ΔE compared to the other methods. On the other hand, the control and US10 samples did not show significant changes in color change. The color degradation during ET pretreatment of petals may be due to non-enzymatic browning and pigment oxidation and anthocyanin structure degradation^70^. Furthermore, US30 and ET/US pretreatment helped minimize degradation index and color change compared to other pretreated samples.

### Rehydration ratio (RR)

**Fig. 8:**
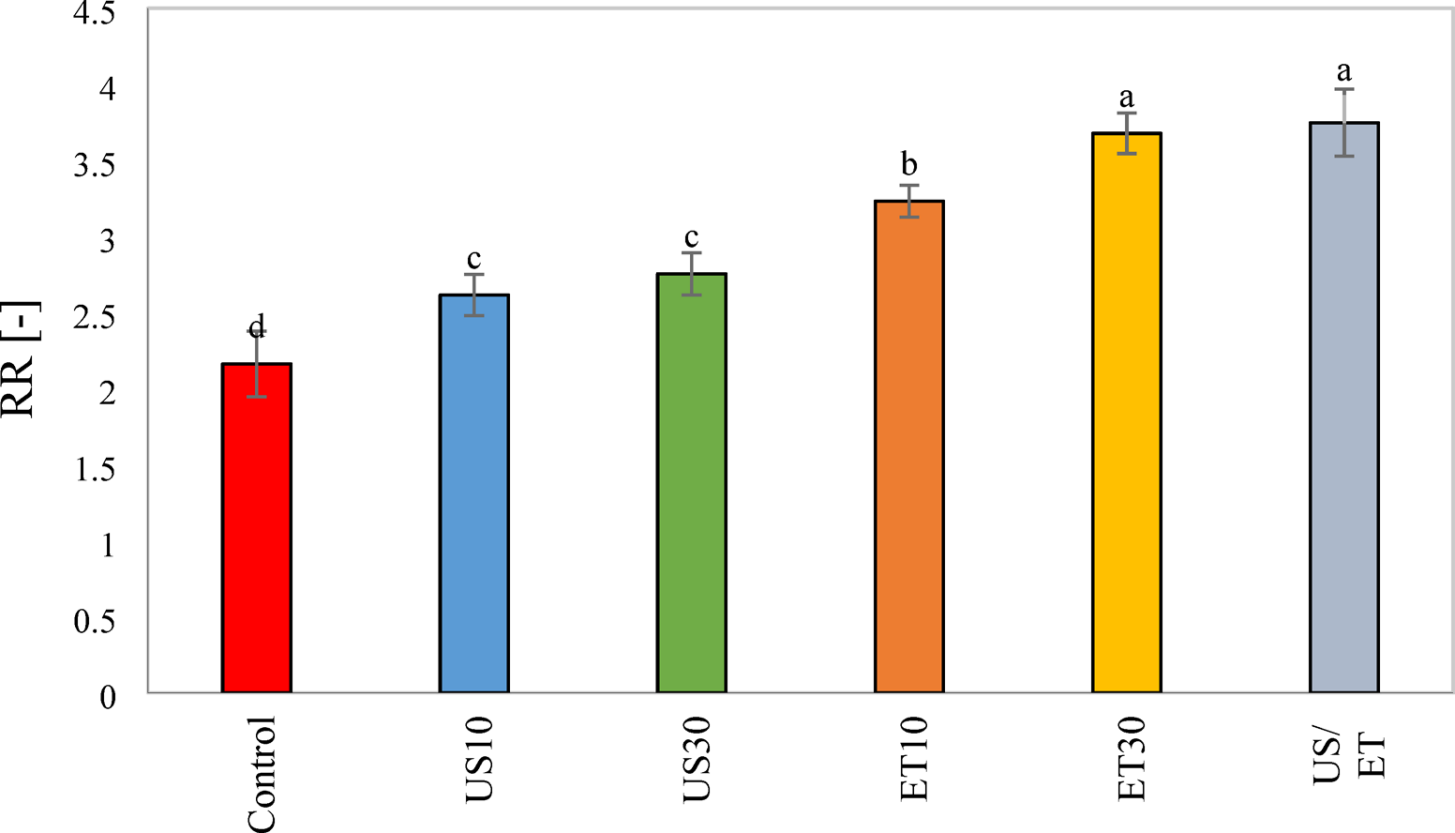
Rehydration rate of the untreated and pretreated rose petal.

The effect of ET, US and ET/US pretreatments before hot air drying on rehydration ratio (RR) were also considered. There was a significant difference between the RR in different pretreatments with the control sample (p < 0.05). All pretreatments had a higher RR compared to the control sample. Ethanol and ultrasound has been shown to improve rehdration in previous literature, including pumpkin^71^ and scallion^72^ with ethanol, and purslane^27^ and ginger^73^ with ultrasound and grape^44^, carrot^31^ and pumpkin^48^ with ethanol/ultrasound. The control had the lowest RR, probably due to structural changes from prolonged drying. US pretreatment (US10 and US30) significantly affected the RR (P < 0.05). However, the variation obtained between the samples dried was not notable at different sonication durations (Fig. 8). In contrast, ET pretreatment increased the rehydration ratio, with ET30 achiveing the highest water absorption. However, US-treated samples (US10 and US30) had lower RR than ET-treated (ET10 and ET30). In the US bath, the product cells swell due to the filling with water, so during the drying process of the product, water has to be removed from the cells, which greatly damages the cell structure. According to the results of Fig. 8, the RR of ET/US was similar to that of the longer ET pretreatment (E30). However, the highest value was recorded in ET/US. In fact, ET/US had a higher rehydration capacity compared to the control (40.4%), US10 (30.1%), US30 (27.9%), ET10 (13.8%) and ET 30 (2.12%). The improvement in rehydration capacity in ET/US was attributed to the structural changes in tissues and cells caused by it, which facilitate water transport and/or retention. This fact has been previously reported in the rehydration of peach^74^, pumpkin^50^ and grape^56^. These results of improved rehydration with ethanol and ET/US pretreatments could be interesting for purposes such as compounds of interest in food matrices.

### Antioxidant, phenolic and flavonoid content

**Fig. 9:**
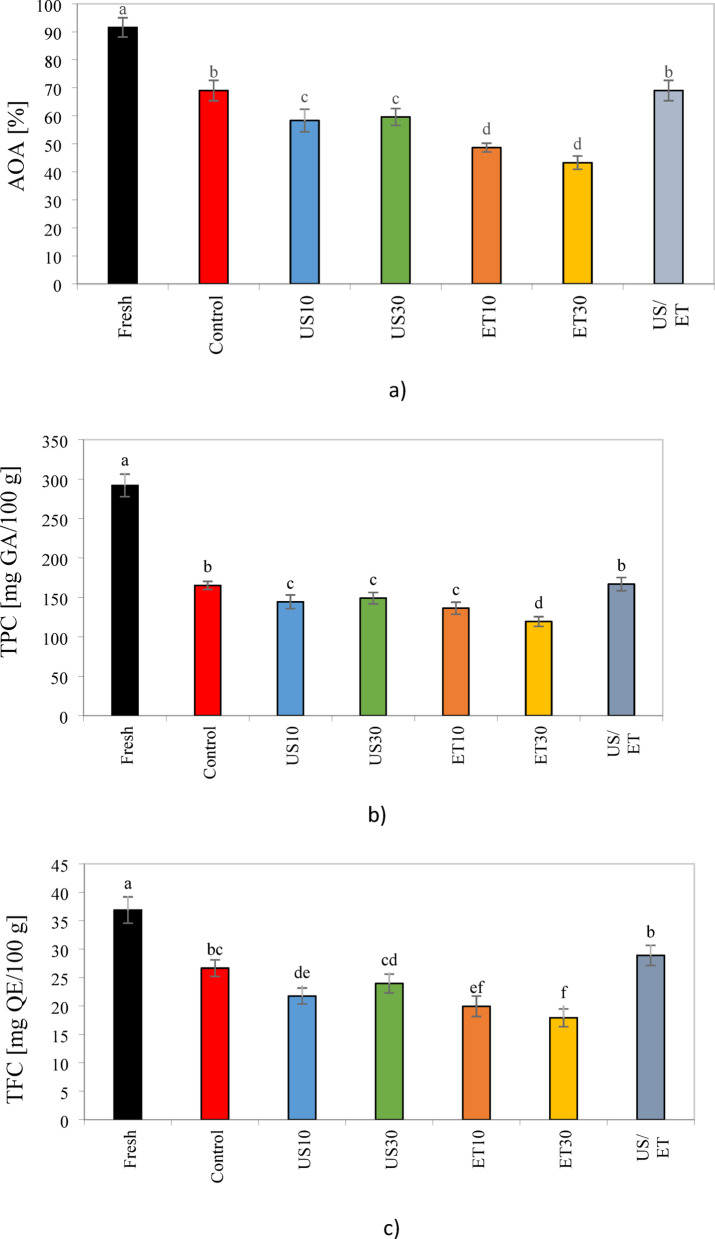
Antioxidant activity (**a**), Total phenol content (**b**) and Total flavonoid content (**c**) of the untreated and pretreated rose petal.

Average antioxidant (AOA), total phenolic content (TPC), and total flavonoid (TFC) for fresh and dried rose petals are presented in Fig. 9 (AOA- a, TPC- b and TFC- c). As can be seen in Fig. 9, AOA, TPC and TFC were significantly reduced by these drying pretreatments compared to control samples. The average AOA, TPC and TFC values of fresh rose were 91.59%, 289.95 mg GA/100g, and 38.65 mg QE/100g, respectively. As expected, AOA, TPC and TFC were reduced for all drying treatments compared to the fresh sample. In a study, Barani et al.^38^ recorded similar results and reported the loss of TPC and TFC in rose petals dried by infrared and ultrasound. The greatest losses occurred in ET30-treated (P < 0.05): AOA decreased by24.7%, TPC by 13.66%, and TFC by24.3%. The resaon of this reduction could be due to ethanol causing further and irreversible oxidationand consequently further cell wall degradation^61^. Kian-Pour et al.^52^ similarly reported a reduction in TPC and AA in beetroot samples pretreated with ethanol compared to untreated samples. In another study, Miano and Rojas^75^ stated that immersing samples in ethanol may extract components that lead to their easy degradation, thus reducing TPC and AOA. da Cunha et al.^76^ found that melon samples pretreated with ethanol at concentrations of 50 and 100% significantly decreased (p < 0.05) before hot air drying.

Additionally, US waves can alter the microstructure of petals by disrupting cell walls and vacuoles, thereby releasing bioactive compounds into the pre-treated medium. The values of AOA, TPC and TFC obtained by US pre-treatment was lower than that of the control and ET/US combined samples, but higher than that of ET pre-treatment. Amanor-Atiemoh et al.^55^ showed an increase in phenolic compounds in apples exposed to US waves compared to ethanol. They stated that the disruption of the cell wall due to acoustic cavitation by creating mechanical pressure on the sample causes the release of TPC and TFC compounds. Also, Aadil et al.^77^ pointed out that additional OH groups during cavitation can produce more phenolic compounds. Similar results were reported by Ren et al.^16^ and Tepe^20^, who reported higher retention of AOA, TPC, and TFC compounds as a result of the use of ultrasound compared to ethanol for ginger and apple juice, respectively.

On the other hand, as shown in Fig. 9a, b, and c, it was observed that the ET/US combination significantly (P < 0.05) improved AOA, TPC and TFC parameters compared to other pretreatments. Therefore, the ET/US combination is the best pretreatment to maintain most of the mentioned properties. These findings were consistent with Granella et al.^35^. They also indicated that the higher retention of AOA and TPC compounds in banana slices with ET/US was due to their shorter drying time and lower thermal degradation. Also, Osae et al.^25^ and Amanor-Atiemoh et al.^55^ attributed the retention of AOA, TPC and TFC compounds to the enhancing effect of US and ET and emphasized that the synergistic effect of ET/US leads to increased extraction and increased mass transfer of phenolic compounds. Furthermore, according to Ren et al.^16^ and da Silva et al.^56^, the osmotic dehydration effect of ET and the increased mass transfer and extractability under US due to the synergistic effect of ethanol/ultrasound can preserve AOA, TPC and TFC compounds better than other pretreatments.

### Essential oil yield (EOY)

**Fig. 10:**
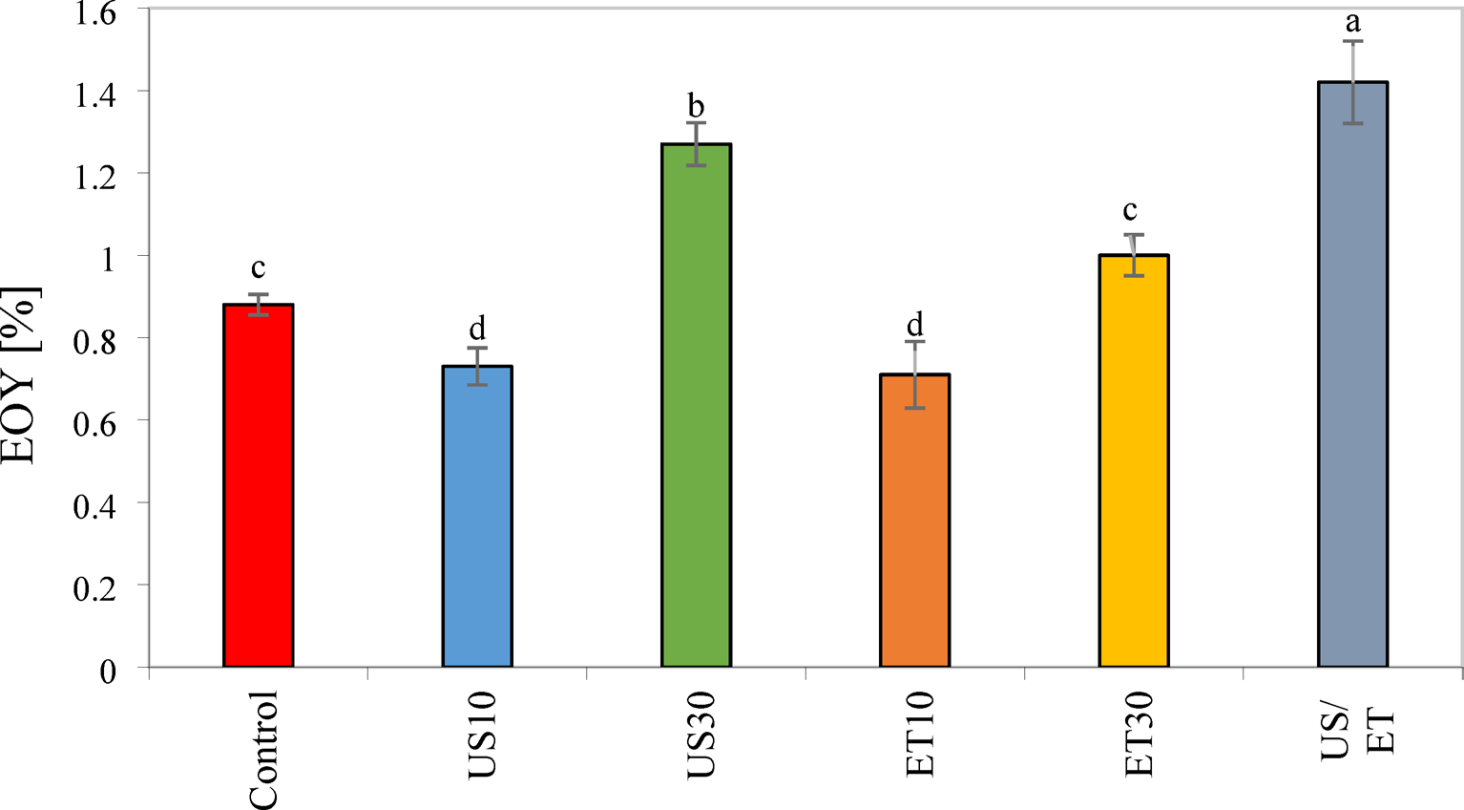
Essential oil of the untreated and pretreated rose petal.

Regarding the effect of different pretreatments on the essential oil yield (EOY) parameter of rose petal, the results show that all pretreatments have a significant effect on EOY (p < 0.05). Figure 10 shows the average EOY extracted from dried roses for the pretreatments applied before hot air drying. Based on the results obtained, the general trend of changes in EOY, the lowest value (0.75%) was observed in ET10, while the highest EOY value was recorded in ET/US (1.31%). According to the results of Fig. 10, US10 reduced EOY by 11.3% less than the control samples, while US30 increased EOY by 39.7%. Therefore, longer US durations enhanced EOY significantly (P < 0.05). due to the accelerating mass transfer and reducing drying time by creating fine channels inside the product. Therefore, the use of simultaneous ultrasound reduced the process time and increased the amount of essential oil. Samani et al.^78^ reported that the use of US increased the EOY in *Satureja bachtiarica* samples.

Regarding drying rose petals with ethanol pretreatment (ET10 and ET30), a significant decrease (14.7%) in EOY was observed with ET10 compared to the control sample. In ET10 samples, more volatile compounds may evaporate along with moisture from the surface of the petals. However, the combined ET/US pretreatment increased the EOY by 51.1%, 70.5%, 8.1%, 77.3%, and 0.40% in hot air drying compared to the control methods, ET10, ET30, US10, and US30, respectively. In ET/US treatment, due to the synergistic effect and shorter drying time, the oil glands may be less damaged. Albuquerque et al.^9^ observed that combined ET/US pretreatment for 12 min resulted in higher EOY than other treatments in drying *Schinus terebinthifolius Raddi*.

## Conclusions

The present study demonstrated that applying non-thermal pretreatments, particularly the combined ethanol and ultrasound method (ET/US), substantially improved the drying efficiency and quality of rose damascena petals. All pretreatments improved the drying kinetics and moisture diffusion of rose petals in comparison with reference samples, with the effect of ethanol/ultrasound pretreatment being greater than other pretreatments due to the synergistic effect (Marangoni and cavitation phenomena). In addition, ethanol/ultrasound pretreatment reduced the drying time of rose petals by 52.38%. The maximum and minimum reduction in drying energy consumption was observed in ethanol/ultrasound and US10 treatments of 64% and 14.2%, respectively. Among the different pretreatments, the lowest water activity and the highest moisture diffusion were obtained for ethanol/ultrasound. While the lowest color changes were obtained for US30. Also, ultrasound and ethanol pretreatments alone reduced TPC, AOA, and TFC compared to control samples, while their values increased with ethanol/ultrasound pretreatments. These results can be attributed to the synergistic mechanisms of ethanol-induced membrane disruption and ultrasound-assisted cavitation, which together enhance mass and heat transfer. The study introduces ET/US as a promising, scalable technique for the drying of sensitive medicinal and aromatic plants. Future work may explore its industrial-scale applicability, economic feasibility, and impact on a wider range of botanical materials.

## Data Availability

All data and materials are available upon reasonable request from the corresponding author.

## Abbreviations

A: Cross-sectional area of drying (m^2^)
A_c_: Absorbance value of the control
A_d_: Absorbance value of the dried sample solution
AOA: Antioxidant activity
C_pa_: Specific heat capacity of ambient air (J/kg K)
D_eff_: Effectivie moisture diffusivity (m^2^/s)
DR: Drying rate (kg water/(kg dry matter min))
EOY: Essential oil yield (%).
E_w_: EO weight
L: Thickness of the material
M_w_: Sample mass (kg)
M_t_: Moisture content at time t
Mi: Indicates the initial moisture content (wet basis)
M_o_: Denotes the mass of the *S. officinalis leave* desiccated at time t (kg)
M_e_: Equilibrium moisture content
M_w_: Mass after drying (kg)
m_r_: Mass of samples after rehydration (g)
m_d_: Quality of the dehydrated product (g)
MC: Moisture content at time t
MR_pre,i_: Predicted moisture ratio
MR_exp,i_: Experimental moisture ratio obtained from the drying experiments
M_f_: Mass after drying (kg)
N: Number of experimental data
RMSE: Root mean square error
R^2^: Coefficient of determination
RR: Rehydration ratio
SET_CON_: Specific energy consumption during hot dryer pretreatment (MJ/kg)
SET_US_: Specific energy consumption during US pretreatment (MJ/kg)
t: Drying time (min)
t_c_: Drying time needed to the samples reach a moisture 10% w.b.
TEC: Total Specific energy consumption (pretreatment + hot air) (MJ/kg)
t_p_: Time of US pre-treatment (s)
v: Air velocity (m/s)
V: Volume of water (L)
W: US volumetric power (W/L)
W_s_: Dry matter weight
z: Number of parameters in the model
a*: Changes from − 100 (red) to + 100 (green)
b*: Whereas the variation range b* is from − 100 (blue) to 100 + (yellow)
L^*^: Brightness of the sample that varies in the range of 0 to 100
Δt: Temperature difference between the ambient air and drying air
χ^2^: Chi-square
ρ: Ambient air density
